# On the Population Dynamics of Junk: A Review on the Population Genomics of Transposable Elements

**DOI:** 10.3390/genes10060419

**Published:** 2019-05-30

**Authors:** Yann Bourgeois, Stéphane Boissinot

**Affiliations:** New York University Abu Dhabi, P.O. 129188 Saadiyat Island, Abu Dhabi, UAE; yb24@nyu.edu

**Keywords:** transposable elements, population genetics, selection, drift, coevolution

## Abstract

Transposable elements (TEs) play an important role in shaping genomic organization and structure, and may cause dramatic changes in phenotypes. Despite the genetic load they may impose on their host and their importance in microevolutionary processes such as adaptation and speciation, the number of population genetics studies focused on TEs has been rather limited so far compared to single nucleotide polymorphisms (SNPs). Here, we review the current knowledge about the dynamics of transposable elements at recent evolutionary time scales, and discuss the mechanisms that condition their abundance and frequency. We first discuss non-adaptive mechanisms such as purifying selection and the variable rates of transposition and elimination, and then focus on positive and balancing selection, to finally conclude on the potential role of TEs in causing genomic incompatibilities and eventually speciation. We also suggest possible ways to better model TEs dynamics in a population genomics context by incorporating recent advances in TEs into the rich information provided by SNPs about the demography, selection, and intrinsic properties of genomes.

## 1. Introduction

Transposable elements (TEs) are repetitive DNA sequences that are ubiquitous in the living world and have the ability to replicate and multiply within genomes. Since their discovery, TEs have proven to be of paramount importance in the evolution of genomes, shaping their architecture, diversity, and regulation [1,2,3,4]. Given their abundance, the precise quantification of the evolutionary forces and mechanisms that condition their polymorphism and eventual fixation or loss in natural populations is needed.

The theoretical and practical tools provided by population genetics have been crucial to better understand how stochasticity and selection shape TEs dynamics (e.g., [2,5,6,7]). The first demographic models specifically designed for the analysis of TE polymorphisms were already developed in the 1980s, incorporating transposition and excision rates, effective population size, and purifying selection [4]. Despite this early interest, the investigation of TEs’ dynamics in natural populations faded between 1990–2000 [8]. While the precise mechanisms underlying the activity and copy number of TEs have been the topic of many early studies, relatively little attention has been paid to their microevolutionary dynamics in the genomic era, when the focus has been on comparative genomics and on analyses at deeper evolutionary scales. This is mostly explained by the sequencing technologies that have, until recently, produced rather short sequencing reads, which prevent the accurate identification of TE insertions. Instead, most population genomics studies have focused on variation regarding single nucleotide polymorphisms (SNPs). The growing availability of whole-genome resequencing data, as well as the development of new computational tools, has revived the interest of the evolutionary genomics community for the analysis of TE polymorphisms [9,10].

Early reports on the propagation of TEs demonstrated a deleterious effect of their activity. This work, which was mostly based on the investigation of TE polymorphisms in *Drosophila* populations, presented this type of variation as neutral or deleterious [11], and subsequent studies have tried to explain the allele frequency spectrum of TEs within this framework [5,12]. However, TEs can dramatically modify phenotypes, for example by triggering epigenetic mechanisms, by modifying gene expression, or by being a source of ready-to-use functional motifs [13,14]. Thus, TEs can potentially be recruited in adaptive processes and rise in frequency due to positive selection. It remains unclear how the abundance and frequency of TEs are controlled by the host, and to what extent they can become the target of positive selection [9]. In addition, understanding the dynamics of TEs requires jointly studying the host demography, adaptation, and mechanistic views of genome architecture, regulation, and coevolution. This will be crucial if we want to quantify the importance of TEs in adaptive processes and the evolution of species. Here, we summarize the current state of the literature on TEs’ evolution at microevolutionary scales, but we also propose possible methodologies to jointly study TEs and traditional markers such as SNPs.

## 2. Transposable Elements: Classification and Mechanisms of Transposition

“Transposable elements” is an umbrella term that covers a wide diversity of DNA sequences that have the ability to move from one location of a genome to another location. Besides being mobile, these sequences don’t have much in common, and they differ considerably in sequence, structure, length, base composition, and mode of transposition. A number of excellent reviews are available on TE diversity (among those, we refer the reader to [15,16,17]), and we provide here a short synthesis of what is known. TEs are broadly classified into two classes: class I elements (or retrotransposons), which are mobilized by the reverse-transcription of an RNA intermediate, and class II elements (DNA transposons), which use a DNA intermediate. Retrotransposons are further divided into long terminal repeats (LTR) and non-LTR retrotransposons, based on the presence of long terminal repeats (LTR). LTR retrotransposons, which include the *copia* and *gypsy* elements, are mobilized by a process similar to retroviruses. The RNA is reverse-transcribed in the cytoplasm into a double-strand cDNA, which is inserted back into the genome by an integrase. Non-LTR retrotransposons, which include the Long Interspersed Nuclear Elements (LINEs) and *Penelope* elements, are mobilized by a mechanism termed target-primed reverse transcription, where the RNA is reverse-transcribed at the site of insertion [18]. The reverse transcriptase of non-LTR retrotransposons can also act on other transcripts and is responsible for the amplification of non-autonomous elements (also called Short INterspersed Elements, or SINEs), which can considerably outnumber their autonomous counterparts [19]. Class II elements include elements that use a cut-and-paste transposition, such as the *hAT* and *mariner* elements, or elements that have a circular DNA intermediate (*Helitrons*). Class II elements can also mediate the transposition of non-autonomous copies, which, similar to SINEs, can amplify to extremely high copy numbers.

Since TEs are part of the genome of their hosts, they are transmitted vertically from parents to offspring. However, many elements have the ability to invade genomes horizontally, and the recent sequencing of a large number of eukaryotic genomes revealed that this process is not as uncommon as previously thought. Some elements seem to be more prone to horizontal transfer than others. Non-LTR retrotransposons are transmitted mostly vertically [20,21,22], but some families, such as *RTE*, have been shown to readily transfer across highly divergent taxa, for instance from reptiles to cows [23,24]. The horizontal transfer of LTR retrotransposons is more frequent and seems particularly common in plants and insects [25,26]. Similarly, the horizontal transmission of DNA transposons has been widely documented, and for some unknown reason, some organisms, such as butterflies, bats, and squamate reptiles, seem much more prone to horizontal transfer than others [27,28,29,30,31]. Another case of horizontal transfer occurs when the germline is invaded by retroviruses, which can become stable residents of genomes, keeping the ability to multiply in the genome while lacking infectivity [32,33].

The abundance and diversity of TEs differ considerably among organisms, and the evolutionary mechanisms responsible for these differences remain unclear. The number of TE copies is highly correlated with genome size and can show large variation, even within the same eukaryotic lineage. For instance, among parasitic unicellular eukaryotes, TEs are absent from the genome of *Plasmodium falciparum* [34], while the genome of *Trichomonas vaginalis* is composed of 40% TEs [35]. In plants, ~85% of the maize genome is composed of TEs [36], whereas this number is only ~10% in *Arabidopsis thaliana* [37]. Among vertebrates, the abundance in TEs range from ~6% in the pufferfish to more than 50% in zebrafish and some mammals [1,38]. The diversity of TEs also differs considerably among organisms. For instance, the genome of non-mammalian vertebrates (fish, amphibian, reptiles) typically contains a large diversity of active TEs represented by many families of class I and class II elements, whereas the genome of placental mammals generally harbors a single type of autonomous TE: the LINE-1 (L1) element [1,38,39,40].

## 3. How Population Dynamics and Intrinsic Properties of Genomes Shape TEs Polymorphisms

### 3.1. The Role of Purifying Selection and Demography

**Figure 1:**
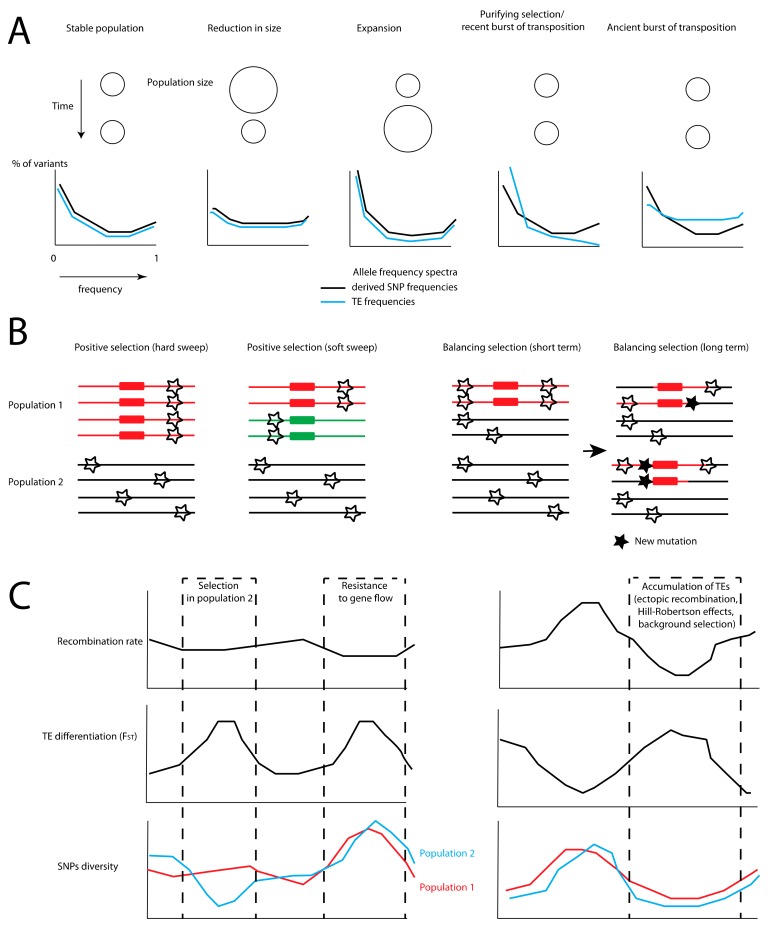
Summary of mechanisms impacting the diversity and frequency of transposable elements (TEs), and their impact on flanking sequences. (**A**) Demographic changes affect the frequency spectra of both TEs and single nucleotide polymorphisms (SNPs) in a similar way, assuming neutrality and a constant rate of transposition. Reductions in effective population sizes should lead to an excess of alleles at intermediate frequencies, while population expansions may lead to an excess of singletons. On the other hand, purifying selection on TEs should lead to an excess of singletons compared to SNPs. Variable rates of transposition may also lead to discrepancies in the spectra between SNPs and TEs. (**B**) TEs involved in adaptation may be detected through their changes in frequencies, but also through the signature left in flanking regions. In the case of positive selection, longer, younger haplotypes should be found nearby positively selected insertions. The similarity of selected haplotypes may be very high in the case of a recent hard sweep, where the insertion is immediately selected and rises in frequency. It may be lower in the case of a so-called soft sweep, where selection either acts after the insertion has already reached an appreciable frequency in the population, or when two insertions with a similar effect on fitness appear at the same time. Positive selection should also result in higher differentiation at the selected locus compared to populations where selection is not acting. On the other hand, balancing selection may lead to signatures of partial selective sweep when it is recent. Since the selected alleles may be maintained through long periods of time, they have more time to recombine and accumulate new mutations than neutral haplotypes, leading to a narrow signature of high diversity. Since alleles under balancing selection tend to introgress into new populations, and have high diversity, low differentiation is expected at these sites. (**C**) Left panel: Given a constant recombination rate, positive and linked selection in a given population (here, a population of two) may increase differentiation and reduce diversity at selected TEs and flanking regions compared to the rest of the genome. On the other hand, if TEs play a role in incompatibilities after secondary contact, a signature of both elevated differentiation and diversity may be expected. Right panel: However, an excess of TEs in regions of reduced polymorphism, higher differentiation, and lower recombination may be caused by different mechanisms such as purifying selection. This can be due to a reduced effective rate of transposition in regions of high recombination due to deleterious ectopic exchanges, and/or because of the larger-scale effect of selection that accelerates lineage sorting and the differentiation of TEs in regions of low recombination.

As for SNPs, the frequency of TE insertions in natural populations is conditioned by the balance among the drift, selection, and migration between demes (Figure 1A). TEs can disrupt genes and regulatory sequences, and thus can negatively affect the fitness of their host. For instance, in humans, several genetic diseases are caused by TE insertions, such as hereditary cancer [41] or haemophilia [42] (for a more exhaustive review, see [43]). This is also exemplified by the extreme rarity of insertions within exons (e.g., in *Drosophila* [44,45] or *Brachypodium distachyon* [46]), compared to intergenic and intronic regions. Thus, it is expected that purifying selection (i.e., selection against deleterious alleles) against TE insertions plays a major role in shaping their frequency in populations. A consequence of purifying selection is that it prevents or delays the fixation of mutations that reduce fitness in a population. This leads to shifts in the derived allele frequency spectrum (AFS), with an excess of derived variants at low frequencies. Many studies have highlighted this effect, using different approaches. Using a diffusion approximation similar to early models of TE evolution [4], Hazzouri et al. estimated the selective coefficient (N_e_s) against an *Ac*-like transposon to range between −50 and −10 in *Arabidopsis arenosa* [47]. In *Drosophila melanogaster*, the selective coefficient against insertions from the *BS* family in an African population was estimated at N_e_s ≈ −4 [48], and was as low as −100 for some TE families [45]. In humans, this coefficient was estimated at N_e_s = −1.9 against L1 retrotransposons [49]. Comparisons of TEs’ frequencies with estimates obtained from coalescent simulations often reveal deviations from purely neutral expectations. This is observed in green anoles [50,51], mice [50], or *Arabidopsis* [7,47], for which TEs display an excess of singletons compared to SNPs, which is consistent with purifying selection. A common point between those studies is that they take into account the demographic history of investigated populations to properly estimate the significance of deviation from neutrality, revealing substantial differences with estimates of N_e_s obtained assuming stable demography [48].

The deleterious effect of TEs can have three causes. First, a cost related to where the element inserts (insertional mutagenesis) can affect the host; the number of disease-causing insertions in humans and other organisms constitute prime examples of this [41,42,43,52]. Second, TEs can produce RNAs or proteins that could be deleterious to the host. For instance, damages induced by the endonuclease encoded by retrotransposons on DNA [53] or the competition of TEs with hosts’ genes for transcription factors [54] may lead to a loss in fitness. Third, ectopic recombination between non-allelic copies can lead to deleterious chromosomal rearrangements. Since the 1980s, the relative importance of each of these three mechanisms has been a matter of debate [4,55,56,57]. However, it has been shown in humans [49], *Drosophila* [57], mouse [50], and anoles [51] that long elements are found at lower frequency in populations than short elements. This suggests that purifying selection acts more strongly against longer copies of elements, and it was shown, in humans, that short elements behave similarly to neutral alleles [49,58]. This pattern could be explained by selection against intact progenitors—which are the longest elements, and the only ones that are capable of producing the RNA and proteins necessary for transposition—or by the ectopic exchange model, since longer elements are more likely to mediate ectopic recombination than shorter ones [50,57,59]. However, selection seems to act against long elements that are not full-length and thus not active, which suggests that the ectopic exchange model plays a preponderant role [50,59]. This model is also supported by the genomic distribution of elements of different length. Long elements tend to be absent from highly recombining regions of genomes [44,60] and accumulate in non-recombining regions such as the human Y chromosome [61,62]. The effect of ectopic recombination will depend on the abundance of elements and the frequency of the insertions. For ectopic recombination to have a substantial effect requires the elements to have reached a copy number threshold so that large families of TEs are more likely to be deleterious than smaller ones [45,57,63]. In addition, heterozygous insertions are more likely to be involved in ectopic recombination because of the lack of an allelic copy on the other chromosome [64]. Thus, elements at low frequency in populations are more likely to be deleterious, since insertions are more likely to be present in the heterozygous state. This suggests that selection against TE insertions may be frequency-dependent, so that the selection coefficient against a specific insertion will decrease when the insertion increases in frequency. Thus, it is expected that rapidly expanding TE families, which are characterized by a high copy number and a majority of insertions in the heterozygous state, are more deleterious than smaller families, where elements are found at high frequency (for instance, after a strong bottleneck effect). These predictions still need to be tested, and this aspect will need to be incorporated in future models of TE evolution. 

Genetic drift is the stochastic variation of allele frequencies across generations due to the finite size of natural populations. The effect of genetic drift will depend on the effective size of populations and their past demographic history. When an effective population size is small, genetic drift can cause large changes in allelic frequency, and may even counteract the effect of selection, so that insertions that would be eliminated by selection in large populations can reach high frequency or even fixation in small populations. The stochasticity induced by demographic events explains a significant amount of TEs’ diversity in natural populations, which is consistent with theoretical models (e.g., [4,65,66]). For example, in *Arabidopsis lyrata*, smaller populations showed an accumulation of TEs at higher frequencies, due to stronger stochasticity and a reduced efficiency of purifying selection in those populations [7,67], and this has been documented across six TE families. In *B. distachyon*, the loss of retrotransposons across genetic clusters is partly explained by recent bottlenecks and demography [46]. In *Drosophila subobscura*, recent bottlenecks explain the high frequencies of the *bilbo* and *gypsy* elements [68]. A recent study demonstrated that TEs’ diversity could be explained by variation in effective population sizes in humans and sticklebacks [50,69], while a joint effect of purifying selection and demography was more obvious in anoles and mice [50,70]. Overall, demography may play an important role in the likelihood for TEs to reach fixation and increase genome size, which is in accordance with the hypothesis that genome size may be directly related to demographic history [71].

### 3.2. Non-Equilibrium between Transposition and Loss

Another important parameter when characterizing TE dynamics is the interplay between the rate of insertion and the rate at which copies are lost from the population. For the sake of simplicity, early models of population genetics applied to TEs have often assumed that these parameters were in equilibrium [66]. However, the frequency of TEs is likely impacted by shifts in this balance. Sudden bursts of transposition can occur, generating a large cohort of insertions with roughly the same age. Such bursts are well-documented in *Drosophila* [72], rice [73], piciformes [74], fish [75], or mammals [28]. On the other hand, hosts defense mechanisms may be triggered by a high level of transposition. This may lead to waves of extinction, with fast drops in the number of functional TE copies in genomes, and ultimately to the complete cessation of transposition. This alteration between periods of proliferation and elimination has sometimes been described as a life cycle [76,77], which results in genealogies between insertions that are quite different from classical turnover expectations [76]. Some stages of this life cycle may be particularly sensitive to high genetic drift, as the stochastic loss of functional copies may lead to the premature loss of transposition compared to large populations [65]. From a population genomics perspective, this non-equilibrium dynamic has a direct impact on the average age of TE insertions in a given population. This affects not only the copy number, but also the frequency spectrum of these insertions. Ultimately, this can generate complications when interpreting discrepancies between the allele frequency spectra obtained from SNPs and TEs, since they may then be explained by a combination of selection and unbalanced ratios between transposition and elimination rates (Figure 1A). For example, an excess of rare insertions may be due to a recent burst of transposition, leading to an excess of low-frequency TEs insertions [78]. Such a signature would be mistakenly attributed to purifying selection in equilibrium models [7,12].

Non-equilibrium explanations for the excess of rare insertions are considered unlikely [5,45] by some authors. Nevertheless, the direct application on TEs of classical population genetics assumptions that rely on constant mutation rates may not be realistic. For example, in *Drosophila*, the frequency spectra of TEs from different families is directly related to each family’s age and their time since inactivation [44]. This may be particularly important for models where little is known about the dynamics of the TEs. To take this issue into account, a test that quantify purifying selection on TEs has been developed [12] that is conditional on the age of elements. However, this age is often overestimated for TE sequences, because of non-equilibrium demography and mutations introduced by transposition errors [12]. Recent advances in modeling may facilitate the deployment of methods that jointly estimate selection and transposition [79].

### 3.3. Transposition and Variable Rates of Recombination

A consequence of selection limiting the proliferation of TEs in genomes is that TEs should be more frequently found in regions of the genome where natural selection and elimination mechanisms are weaker or less efficient. This requires a better quantification of the relationship between the number and the type of TE insertions and genomic features such as recombination, which is often found to be negatively associated with TE content [60,80]. Regions of low recombination tend to be associated with a lower gene content, which reduces the likelihood for an insertion to be strongly deleterious. Selection is more likely to remove TE insertions in regions of high recombination, since more frequent ectopic recombination should increase the likelihood of deleterious chromosomal rearrangements [56]. In addition, TE silencing is often associated with epigenetic modifications that are negatively associated with recombination [81,82]. Another mechanism is Hill–Robertson interference. Competition between haplotypes harboring different deleterious TE insertions may reduce the efficiency of selection, similar to a reduction of local effective population sizes that enhance the impact of genetic drift in regions of low recombination [83,84]. Ultimately, this may lead to the fixation of TEs through the process of Muller’s ratchet, where low recombination prevents the persistence of a haplotype without any insertion, increasing mutational load [56]. However, this latter effect is more likely for TEs in regions of extremely low recombination [56]. The position of recombination hotspots varies across species [85], which can be an alternative explanation to divergent selection when interpreting variation in TE frequencies between species and populations.

Recent studies of recombination landscapes have improved our understanding of TEs dynamics. The expected negative correlation between TEs and recombination rates has been observed for LINEs in humans [59,62], mice, and rats [86]. In *Drosophila*, there is evidence that both reduced gene content in regions of low recombination and ectopic recombination shape the frequency of TEs along the genome [87,88]. However, the insertion process itself varies between different TE families, and may be responsible for variation in abundance and frequency along chromosomes. Indeed, a more detailed examination of the correlation between TEs and recombination shows a heterogeneous pattern, with some TE families [89] and endoviruses [90] found more frequently near recombination hotspots. The same pattern is observed near recombination hotspots in *Ficedula*, which is possibly due to the shared preference of recombination and transposition machineries for open chromatin [85]. A preference for high-recombining regions has also been shown for DNA transposons (but not non-LTR elements) in *Caenorhabditis elegans* [91]. This may be due to the cut-and-paste mechanism of transposition that takes advantage of the double-stranded breaks that initiate recombination events. Another possible explanation lies in the negative correlation between the age of TEs and the recombination rate, suggesting that a long-term effect of recombination is needed to remove TEs from genomes. Overall, this suggests that previous demonstrations of a negative correlation between TE content and recombination rate need to take into account the properties and histories that are specific to each TE family [60,91].

**Figure 2:**
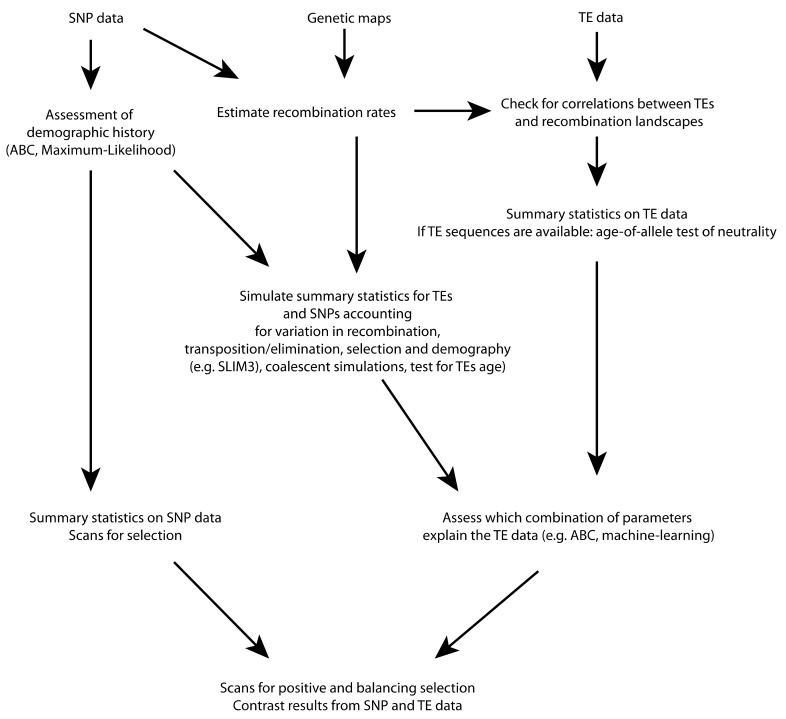
A possible analytical pipeline for population genomics of TEs, highlighting some promising methods. Genetics and genomics may provide information about the intrinsic properties of genomes (e.g., recombination maps) and extrinsic processes such as demographic changes and selection. This information may then be used to build neutral expectations about both TEs and SNPs. Contrasting the observed statistics for TEs (e.g., frequencies, length, properties of flanking regions) with simulations may facilitate the quantification of the mechanisms that act on their diversity.

Until recently, most theoretical works on TE dynamics have considered constant recombination rates [56]. The emergence of new simulation tools that can simultaneously incorporate the intrinsic properties of the genome and the evolutionary history of populations may be valuable to disentangle the effects of demography, selection, recombination, and the transposition process of TEs (Figure 2). A promising method is SLIM3 [79], which is able to simulate TEs as well as flank genomic fragments under any arbitrary complex demographic scenario, and can also incorporate variations in transposition rates due to thresholds in abundance or any other feature deemed useful by the user. Then, contrast between simulations and observed data may be performed to quantify the dynamics of TEs, for example through approximate Bayesian computation (ABC) [92] approaches (see [50] for an example).

### 3.4. Coevolutionary Dynamics

Coevolution between TEs and their hosts is a crucial aspect that shapes TE diversity and impacts the likelihood for insertions to reach high frequencies. Understanding the distribution of TE polymorphisms across genomes and populations requires a better quantification of the mechanisms behind TEs silencing [93]. Refining the timescale of coevolution between TEs and control mechanisms would provide important insights about constraints on the transposition rate. Such knowledge would improve our models of transposition for specific TE families.

Hosts use many mechanisms to control the proliferation of TEs within their genomes (see [94] for an exhaustive review in humans). An important example is the APOBEC enzymes. APOBEC3 proteins inhibit endoviruses by editing dC residues to dU during reverse transcription. This increases the rate of G to A mutation, and ultimately results in the inhibition of transposition. They are also inhibitors of reverse transcription, making them efficient against LINEs and other retrotransposons [95]. Variation in the sequence and structure of APOBEC genes seems to be directly related to their efficiency in controlling TEs [96,97]. There is already evidence that APOBEC proteins act in specific ways on TEs from different families across vertebrates [97]. In vertebrates, epigenetic modifications such as methylation [98] and histone modifications [99] may be responsible for controlling TEs by limiting their expression. In rice, mutants at a chromomethylase, *OsCMT3a*, cannot methylate TEs, and display a burst of transposition [100]. Finally, another control mechanism lies in small RNA pathways, by which TEs RNA is recognized and eliminated. In fruit flies, two main mechanisms regulate TE activity: siRNA/*Dicer* [101] and piRNA [102,103]. Therefore, further refinements of models of TEs’ evolution would benefit from the knowledge of the spatial repartition of methylated regions and other control mechanisms that are specific to the host. A promising approach lies in simulations and model-fitting incorporating demography, selection, and control mechanisms to test expectations about TE dynamics. For example, a recent simulation study showed that large, non-recombining clusters of piRNAs are more efficient at trapping TEs and preventing invasions [104]. Transposition rates and population sizes mostly influenced the length during which TEs were active, but not the final amount of TE insertions [104]. Combining experimental evolution with modeling may provide better resolution on the coevolutionary process; an example is provided in [105]. In this work, the authors investigated how synergies between RNAi and methylation pathways effectively controlled TE proliferation, using a set of ordinary differential equations describing transposition, elimination, methylation, and RNA interference. By reanalyzing the expression and transposition of the *Evade* element in two *A. thaliana* inbred lines, they could show that small amounts of RNAi were enough to initiate methylation and silencing. According to the model, the retention of methylated TEs prevented reamplification more efficiently than elimination. Although these models may benefit from further refinements by incorporating unstable demography or linked selection to be broadly applicable, they already provide a solid conceptual and methodological basis.

Importantly, this dynamic implies that there is a coevolution between the different components of the genome, which may have an impact on the diversity of hosts’ defense genes. Scanning the genome for loci that display correlation between their diversity and the number of TE families found in the host may be a way to identify which genes in a pathway are of primary functional importance. There are signatures of fast adaptive evolution at genes that are involved in RNA interference in Drosophila [106], with recent selective sweeps encompassing genes from the piRNA pathway [107]. Another compelling example of coevolution is found in primates, where two zinc-finger genes, *ZNF91* and *ZNF93*, evolved rapidly to prevent the expansion of SINE and LINE elements [108]. Besides the need for a more comprehensive understanding of the pathways involved in TEs regulation, there is a need for further investigation in a population genetics context. For example, are demographic fluctuations such as bottlenecks responsible for a relaxation of selective pressures at defense genes that may explain bursts of transposition? Is there a link between diversity at defense genes associated with speciation and environmental adaptation? 

## 4. Transposable Elements as a Source of Adaptation

### 4.1. Evidence for Positive Selection on TEs and SNPs

Identifying TEs that are under positive selection and therefore rise to high frequency in populations is an exciting alley for research in population genomics. However, detecting positive selection is a challenging task even for traditional markers such as SNPs [109]. TEs idiosyncrasies must also be taken into account, since bursts of transposition or insertion bias due to recombination also shape their diversity. Many TEs have been domesticated by hosts genomes over long evolutionary time scales, leading to the emergence of novel cellular functions through the recruitment of TE-derived coding sections or *cis*-regulatory domains [110]. For example, the RAG genes that are involved in the recombination process of antibodies in jawed vertebrates [111,112] originated from a domesticated *Transib* element [113]. Whole TE families may be domesticated by a host. For example, in *Drosophila*, three non-LTR retrotransposons (TART, TARHE, and HeT-A) preferentially transpose in telomeres and prevent their shortening [114], although their domestication is likely incomplete [115]. TEs are also important for the stability of centromeres during replication [116], and might be involved in speciation. For example in rice, recent insertions of both class I and class II transposons are responsible for the accelerated differentiation of centromeres between three cultivated species and subspecies [117].

Bursts of transposition are known to occur in organisms put under stressful conditions [118], which may be subsequently recruited by the host for rapid adaptation [2,119]. For example, the increased transposition of BARE-1 may be adaptive and is associated with higher elevation and dryness in natural populations of the wild barley [120]. A burst of transposition is associated with the adaptive radiation of *Anolis* lizards. This has led to an increase in TE insertions within the *HOX* genes clusters compared to other vertebrates, which may be linked to the outstanding morphological diversity in these lizards [121]. In maize, the expansion of Helitrons might have been associated with positive selection over 4% of these elements [122]. Some *Helitrons* subfamilies can capture gene fragments. The survival rate of these elements was correlated with the length of genetic inserts, which might enhance their adaptive potential. 

TEs can provide a selective advantage and quickly modify phenotypes, for example by triggering epigenetic mechanisms and enhancing gene expression due to the insertion of a TE promotor [13,123]. A recent example includes the genetic determinism of the industrial melanism trait in peppered moth, which is associated with a TE insertion in the *cortex* gene [124]. In *Drosophila*, there is evidence that TEs may be recruited in adaptation to temperate environment, pesticides [125,126], development [127], or oxidative stress [128,129]. The same insertion may have both positive and negative effects on fitness [127,130], which may prevent fixation due to the associated cost of selection. In humans, analyses based on TE frequencies in 15 populations sampled across Europe, Asia, and Africa highlighted candidate TEs for adaptation that might be responsible for change in gene expression [131]. However, we note that unlike recent studies in *Drosophila* [129], this study focused primarily on TE frequencies, and did not examine signatures of selection in flanking regions, and used a relatively simplistic model of human demography. Importantly, similar to traditional markers such as SNPs, the effects of past demography may mimic expected signatures of selection. For example, in *D. melanogaster,* latitudinal variation in North America and Australia was partly explained by past admixture between African and European populations [6]. Overall, the way that TEs are recruited by the host—either through the recycling of TE-derived coding regions (e.g., *RAG* genes), because of the repeats themselves (e.g., TART) or because of regulatory effects (cortex in peppermoth, [132]—the candidate genes in humans [131]) still need to be quantified. 

### 4.2. Quantifying Positive Selection on TEs 

A promising approach consists in the joint analysis of TEs and SNPs to detect candidate insertions for positive selection (Figure 1B,C and Figure 2). SNPs can be used to build neutral demographic models and allele frequency spectra that are expected under neutrality [7,51]. Variation in allele frequencies across populations can be used to detect insertions displaying high differentiation driven by positive selection [10,133]. A common bias in these approaches is that background selection can also lead to unusual allele frequency spectra and patterns of differentiation due to stronger drift in regions of low recombination. A possible way to overcome this issue and identify loci that are truly under positive selection consists of performing genome-wide association with environmental or phenotypic features [109]. Other approaches based on linkage disequilibrium (LD) can help identify insertions that are associated with long haplotypes, and are therefore more likely to be under recent positive selection. The distribution of haplotypes’ length may provide useful information to estimate the age of an insertion (see for example [124]). A number of tests, including iHS, XP-EHH, and H2/H1 statistics or nSL [134,135,136,137], can be used on datasets combining TE insertions and SNPs. 

Other approaches that directly link environmental and phenotypic variation to SNPs may be applied to TEs as well. Methods that track association between allele frequencies and environmental features across populations are increasingly powerful (e.g., BAYPASS [138]). Classical genome-wide association analyses (GWAS) at the scale of individual phenotypes are also a good way to better link TEs variation with relevant ecological mechanisms that may shape diversity. Other potentially fruitful approaches have been developed that facilitate the joint inference of demography and selection and make a better use of whole-genome information. Those include ancestral recombination graphs (ARGs) inference [139], approximate Bayesian computation (ABC) [92], and machine learning [140]. ARGs inference reconstructs coalescent and recombination landscapes along genomic fragments, and is useful to quantitatively estimate the time since selection and completeness of selective sweeps. However, this inference is computationally intensive and unpractical for very large datasets [139]. ABC and machine learning are faster approaches that use summary statistics computed across genomic windows to classify them as selected or not. These approaches allow combining multiple tests for selection such as the ones described above. Then, expectations for these statistics can be obtained by simulations under the hypothesis of selection or neutrality, and algorithms can be trained to classify windows as more or less likely to contain selected sites [141,142]. This type of approach has the advantage of directly including the confounding effects of demography in its implementation, and provides an estimate of false positive and false negative rates. 

A general question in the study of adaptation at the genomic level lies in identifying the origin of beneficial alleles. Selected alleles can have independent mutational origins and rise independently in the frequency in each population, as they provide a selective advantage. Selected alleles might originate from novel alleles that quickly reach high frequency due to their benefit (hard sweep) or from pre-existing standing variation (so-called soft sweeps [143]). At last, an allele initially selected in one population can spread through migration to other populations where it provides a selective advantage. These questions are especially interesting for TEs. For example, biases in transposition due to recombination and coevolution with the host may facilitate the repeated emergence of advantageous mutations in the same genomic regions, ultimately promoting convergent evolution. Methods similar to diploS/HIC [144] may be used to disentangle scenarios of neutrality, selection on de novo mutations (hard sweep), or on standing variation (soft sweep). Another recently developed maximum-likelihood approach, dmc [145], aims at distinguishing between different modes of convergent adaptation at candidate sites for selection, and may be useful to use on candidate TEs for adaptation and flanking SNPs.

### 4.3. Studying Balancing Selection on TEs

Evidence for balancing selection, a type of selection that maintains variation, is still elusive in natural populations, even for SNPs (but see [146] for a discussion of its importance). This type of selection is notoriously difficult to detect due to its very localized effects, especially on long evolutionary time scales. Several recent methods have been specifically developed to detect this type of selection [139,147,148], and may be used on TEs or linked SNPs and haplotypes (Figure 1B). The role of TE insertions in facilitating balancing selection is worth investigating, although neglected [149]. A recent example in a locust is a *Lm1* insertion in the heat-shock protein *Hsp90*, which is found only in the heterozygote state and seems to display latitudinal variation [150]. This insertion is associated with the faster development of embryos, and may control the number of broods that hatch in a year. Instead of directly providing a selective advantage, TEs might facilitate the maintenance of diversity at loci where their expression at the homozygote state would be detrimental, for example at genes of the Major Histocompatibility Complex [151].

### 4.4. Limitations and Future Improvements

A word of caution is needed, since all those approaches are more likely to identify whole genomic regions than specific TE insertions under selection. Therefore, functional validation remains an essential step to identify TE insertions that have a positive impact on fitness [9]. Moreover, several types of selection remain difficult to detect and quantify, such as multi-locus weak selection or balancing selection [109]. However, it is now possible to address such issues, as recent advances in sequencing will allow for the inclusion of large number of individuals in a dataset, and will thus facilitate the narrowing of candidate regions for selection. Low-depth sequencing becomes an interesting way to obtain genotypic information for many individuals [152], and may be associated with the systematic search for transposable elements using state-of-the-art methods such as MELT, which have been shown to perform well when detecting polymorphic variants, even at relatively low sequencing depths [153]. However, other methods are being developed (Table 1), and may be more suited to a specific design, such as pooled whole-genome resequencing. This may be coupled with recent improvements in GWAS such as mixed linear models that have enhanced power to detect the loci associated with relevant phenotypes and polygenic selection [154] using large sample sizes.

## 5. The Role of Selfish Elements in Genomic Conflicts: Impact in Natural Populations

During speciation, populations may diverge and accumulate private combinations of alleles at multiple loci. The disruption of these allele combinations in hybrids may result in lower fitness, which is a process known as Bateson–Dobzhansky–Muller incompatibilities, and prevents the homogenization of gene pools [168,169]. These incompatibilities can emerge when conflicts between selfish elements and the host lead to different coevolutionary mechanisms in isolated populations [170,171,172,173]. Secondary contact between these diverged genomes results in a disruption of the control mechanisms and ultimately the low fitness of hybrids, therefore maintaining differentiated species. TEs may play important roles in these processes (see [174] for a more exhaustive review). A classic example of the hybrid dysgenesis induced by TEs is provided in *D. melanogaster*. In this species, the P-element (a DNA transposon) that expanded recently was probably introduced through horizontal transfer from *D. willistoni* [175,176]. Crosses between females where the P-elements are absent (M females) and P males carrying the element produce progeny exhibiting high mutation rates, chromosomal rearrangements and sterility [177]. This is caused by the deposition of piRNAs in the egg by the females that cannot recognize the P elements provided by the male genome, causing massive expansion. This recent invasion of the P element in *D. melanogaster,* but also in *D. simulans* [178,179,180], highlights the fast dynamic of coevolutionary mechanisms dealing with genomic conflicts and how they can lead to speciation.

Repeated elements are associated with DNA-binding proteins that shape the chromosome organization. There is evidence for the rapid reorganization of these repeats between closely related species (e.g., in rice, [117]) that shape heterochromatin repartition and ultimately disturb the meiotic process in hybrids. Since TEs are associated with major structural changes and variation in repeat content, they may play an important role in meiotic drive, where driver elements rise in frequency by distorting meiosis [173]. Their abundance and high turnover on sex chromosomes (among other repeats) also suggests that TEs may play an important role in the process of speciation and Haldane’s rule, which states that in hybrids between incipient species, the sex that is most likely to display reduced fitness is the heterogametic one [181]. Moreover, TEs can be responsible for gross chromosome rearrangements due to unequal recombination between TE copies [55], which may explain the fast divergence in karyotypes and ultimately speciation (see [182] for a review). TEs may also play a role in dosage compensation between males and females, as demonstrated for a domesticated *Helitron* element in *Drosophila miranda* [183]. In this species, a succession of neo-X chromosomes appeared in the last million years. Gene expression is upregulated by twofold in males by the male specific lethal (MSL) complex that targets an ~21-bp specific sequence harbored by the domesticated element [184]. Domestication of the *Helitron* element occurred each time a new sex chromosome emerged, with a specific motif invading the chromosome and recruiting adjacent genes in dosage compensation. 

How can population genomics contribute to the study of TEs involved in incompatibilities and speciation? First, it remains clear that functional assessments and crosses in controlled conditions may be critical to provide definite proof of the role of TEs in maintaining barriers between species [174]. However, cline theory [185] and the information provided by SNPs can be useful to assess which specific elements may be involved in the speciation process. For example, genomes may be scanned for an excess of private TE insertions in regions of low recombination that resist the gene flow between two species. Since Haldane’s rule predicts that sex chromosomes should be quicker to accumulate incompatibility loci, contrasting the TE content between sex chromosomes and autosomes may also provide evidence for TE-driven incompatibilities. The analysis of SNP and haplotype diversity in regions flanking TEs may also facilitate the interpretation, for example by estimating the age of haplotypes that contain insertions and whether they display evidence of resisting introgression.

Coevolution between TEs and recombination may be important in maintaining divergence between populations (Figure 1C). TEs may drive variation in recombination rates by inducing changes in chromatin conformation; they may also facilitate the suppression of recombination between diverging lineages through their accumulation in low-recombining regions (see [80] for a discussion). This is why when examining the dynamic of TEs after secondary contact, a careful examination of changes in recombination rates along chromosomes and a comparison of correlation between active and inactive families would be recommended [80]. On a related note, variation at genes that shape the recombination landscape may be relevant to assess in association with TEs dynamics. For example, in mammals, *PRDM9* is involved in the fast-evolving positioning of recombination hotspots [186], but it is also involved in hybrid sterility and speciation [173]. Variation at this gene between incipient species may lead to divergent constraints on transposable elements diversity along genomes, which in turn could facilitate the spread of regions of reduced recombination resisting gene flow. 

At last, elements involved in incompatibility may display gradients of association with the environment due to coupling [187], where clines of incompatible alleles drift to match tension zones corresponding to environmental discontinuity. Special care should be taken to identify possible cryptic hybrid zones that can trap incompatible alleles along environmental clines when looking for TEs involved in adaptation to the environment [169,187].

## 6. Future Directions

Recent methodological progresses should prove useful to obtain a better understanding of the dynamics of TEs in natural populations. It is increasingly acknowledged that local variations in mutation and recombination rate, demography, selective sweeps, and linked and background selection have to be integrated into analyses of genetic variation (e.g., [188,189]). All these factors are also likely to explain local variation in TEs density, forcing us to adopt a more integrative approach when studying TEs’ dynamics. Comparisons of simulations-based models are flexible and powerful, and have become increasingly popular in population genomics [92,140]. The challenge with TEs lies in properly simulating the process by which they insert and are removed from genomes, as well as demography and selection. This requires a good preliminary knowledge of the idiosyncrasies of the species and the TEs under investigation. As new methods keep being developed to jointly estimate the effects of demography and selection on genomes, the field of TEs population genomics will move toward more model-based approaches. This will provide quantitative estimates of the forces underlying TEs dynamics.

Another crucial aspect that is still missing for most sequenced species is a high-quality genome assembly. Poor assemblies often omit highly repetitive regions where TEs are more likely to lie. Without proper assembly and annotation, it becomes impossible to perform a near-exhaustive assessment of TE insertions and identification of polymorphisms [9]. This is especially important when investigating the role of repetitive regions in the emergence of incompatibilities. Besides, since the most powerful methods to detect selection use the spatial distribution of allele frequencies and LD, they cannot be used efficiently on highly fragmented genomes. This creates biases; for example, in the Tasmanian devil, poor assembly led to incorrectly assume the inactivation of LINE-1 elements [190]. However, the advent of third-generation sequencing techniques should circumvent this issue and expand the study of TEs to a broader diversity of organisms.

Only a few models are available to study the population genomics of TEs, and drosophilids are clearly over-represented in the field of TE population genetics. This creates a challenge regarding drawing general conclusions about TE dynamics, as well as the relative importance of selection and drift in shaping genomic diversity. The large effective population size of the *Drosophila* species has been hypothesized to facilitate a widespread effect of selection across the genome [189,191], making both demographic inference and the detection of outliers difficult. Besides those on humans, *Drosophila,* and some crops (rice, Arabidopsis, maize), studies remain scarce, with a few studies highlighting the effects of both drift and purifying selection on TE’s diversity in green anoles [51] and birds [192]. As whole-genome assembly and resequencing becomes more affordable, there is hope that more general conclusions about the microevolutionary dynamics of TEs may be drawn. 

## Figures and Tables

**Table 1:** Summary of tools commonly used for transposable elements (TE) detection and analysis. Methods that have been compared on human datasets in [155] are highlighted in bold.

Name of the Method	Purpose	Link	Reference
Popoolation_TE2	TE detection in pooled designs	https://sourceforge.net/p/popoolation-te2/wiki/Home/	[156]
T-LEX2	Detection of polymorphic TEs from short reads	http://petrov.stanford.edu/cgi-bin/Tlex.html	[157]
STEAK	Detection of polymorphic TEs from short reads	https://github.com/applevir/STEAK	[158]
TIDAL	Detection of polymorphic TEs from short reads	http://www.bio.brandeis.edu/laulab/Tidal_Fly/Tidal_Fly_Home.html	[159]
MELT	Detection of polymorphic TEs from short reads	http://melt.igs.umaryland.edu/	[153]
LoRTE	Detection of polymorphic TEs from PacBio sequencing	http://www.egce.cnrs-gif.fr/?p=6422	[160]
ITIS	Detection of polymorphic TEs from short reads	https://github.com/Chuan-Jiang/ITIS	[161]
TEMP	Detection of polymorphic TEs from short reads	https://github.com/JialiUMassWengLab/TEMP	[162]
Mobster	Detection of polymorphic TEs from short reads	http://sourceforge.net/projects/mobster/	[163]
Tangram	Detection of polymorphic TEs from short reads	https://github.com/jiantao/Tangram	[164]
RetroSeq	Detection of polymorphic TEs from short reads	https://github.com/tk2/RetroSeq	[165]
RelocaTE2	Detection of polymorphic TEs from short reads	https://github.com/JinfengChen/RelocaTE2	[166]
McClintock	Combination of several methods into a single pipeline	https://github.com/bergmanlab/mcclintock	[167]
Invade	Population genomics modeling (forward-in-time) incorporating coevolution with piRNA clusters	https://sourceforge.net/p/te-tools/code/HEAD/tree/sim3p/	[104]
SLIM3	Population genomics modeling (forward-in-time)	https://messerlab.org/slim/	[79]

## References

[1] Sotero-Caio C.G., Platt R.N., Suh A., Ray D.A. (2017). Evolution and diversity of transposable elements in vertebrate genomes. Genome Biol. Evol..

[2] Chuong E.B., Elde N.C., Feschotte C. (2017). Regulatory activities of transposable elements: From conflicts to benefits. Nat. Rev. Genet..

[3] Song M.J., Schaack S. (2018). Evolutionary Conflict between Mobile DNA and Host Genomes. Am. Nat..

[4] Charlesworth B., Charlesworth D. (1983). The Population Genetics of Transposable Elements. Genet. Res..

[5] Barron M.G., Fiston-Lavier A.-S., Petrov D.A., Gonzalez J. (2014). Population Genomics of Transposable Elements in Drosophila. Annu. Rev. Genet..

[6] Bergland A.O., Tobler R., Gonzalez J., Schmidt P., Petrov D. (2016). Secondary contact and local adaptation contribute to genome-wide patterns of clinal variation in Drosophila melanogaster. Mol. Ecol..

[7] Lockton S., Ross-Ibarra J., Gaut B.S. (2008). Demography and weak selection drive patterns of transposable element diversity in natural populations of Arabidopsis lyrata. Proc. Natl. Acad. Sci. USA.

[8] Biémont C. (2010). A brief history of the status of transposable elements: From junk DNA to major players in evolution. Genetics.

[9] Villanueva-Cañas J.L., Rech G.E., de Cara M.A.R., González J. (2017). Beyond SNPs: how to detect selection on transposable element insertions. Methods Ecol. Evol..

[10] Hoban S., Kelley J.L., Lotterhos K.E., Antolin M.F., Bradburd G., Lowry D.B., Poss M.L., Reed L.K., Storfer A., Whitlock M.C. (2016). Finding the Genomic Basis of Local Adaptation: Pitfalls, Practical Solutions, and Future Directions. Am. Nat..

[11] Doolittle W.F., Sapienza C. (1980). Selfish genes, the phenotype paradigm and genome evolution. Nature.

[12] Blumenstiel J.P., Chen X., He M., Bergman C.M. (2014). An age-of-allele test of neutrality for transposable element insertions. Genetics.

[13] Morgan H.D., Sutherland H.G., Martin D.I., Whitelaw E. (1999). Epigenetic inheritance at the agouti locus in the mouse. Nat. Genet..

[14] Stuart T., Eichten S.R., Cahn J., Karpievitch Y.V., Borevitz J.O., Lister R. (2016). Population scale mapping of transposable element diversity reveals links to gene regulation and epigenomic variation. Elife.

[15] Tollis M., Boissinot S., MA G.-R. (2012). The evolutionary dynamics of transposable elements in eukaryote genomes. Genome Dynamics.

[16] Craig N., Chandler M., Gellert M., Lambowitz A., Rice P., Sandmeyer S. (2015). Mobile DNA III.

[17] Bourque G., Burns K.H., Gehring M., Gorbunova V., Seluanov A., Hammell M., Imbeault M., Izsvák Z., Levin H.L., Macfarlan T.S. (2018). Ten things you should know about transposable elements. Genome Biol..

[18] Luan D.D., Korman M.H., Jakubczak J.L., Eickbush T.H. (1993). Reverse transcription of R2Bm RNA is primed by a nick at the chromosomal target site: A mechanism for non-LTR retrotransposition. Cell.

[19] Dewannieux M., Esnault C., Heidmann T. (2003). LINE-mediated retrotransposition of marked Alu sequences. Nat. Genet..

[20] Malik H.S., Burke W.D., Eickbush T.H. (1999). The age and evolution of non-LTR retrotransposable elements. Mol. Biol. Evol..

[21] Kordiš D., Lovšin N., Gubenšek F. (2006). Phylogenomic analysis of the L1 retrotransposons in Deuterostomia. Syst. Biol..

[22] Waters P.D., Dobigny G., Waddell P.J., Robinson T.J. (2007). Evolutionary history of LINE-1 in the major clades of placental mammals. PLoS ONE.

[23] Kordis D. (1998). Unusual horizontal transfer of a long interspersed nuclear element between distant vertebrate classes. Proc. Natl. Acad. Sci. USA.

[24] Ivancevic A.M., Kortschak R.D., Bertozzi T., Adelson D.L. (2018). Horizontal transfer of BovB and L1 retrotransposons in eukaryotes. Genome Biol..

[25] Schaack S., Gilbert C., Feschotte C. (2010). Promiscuous DNA: Horizontal transfer of transposable elements and why it matters for eukaryotic evolution. Trends Ecol. Evol..

[26] Bartolomé C., Bello X., Maside X. (2009). Widespread evidence for horizontal transfer of transposable elements across Drosophila genomes. Genome Biol..

[27] Reiss D., Mialdea G., Miele V., de Vienne D., Peccoud J., Gilbert C., Duret L., Charlat S. (2019). Global survey of mobile DNA horizontal transfer in arthropods reveals Lepidoptera as a prime hotspot. PLoS Genet..

[28] Pace J.K., Feschotte C. (2007). The evolutionary history of human DNA transposons: Evidence for intense activity in the primate lineage. Genome Res..

[29] Thomas J., Schaack S., Pritham E.J. (2010). Pervasive horizontal transfer of rolling-circle transposons among animals. Genome Biol. Evol..

[30] Gilbert C., Hernandez S.S., Flores-Benabib J., Smith E.N., Feschotte C. (2012). Rampant horizontal transfer of SPIN transposons in squamate reptiles. Mol. Biol. Evol..

[31] Novick P., Smith J., Ray D., Boissinot S. (2010). Independent and parallel lateral transfer of DNA transposons in tetrapod genomes. Gene.

[32] Ribet D., Harper F., Dupressoir A., Dewannieux M., Pierron G., Heidmann T. (2008). An infectious progenitor for the murine IAP retrotransposon: Emergence of an intracellular genetic parasite from an ancient retrovirus. Genome Res..

[33] Gifford R., Tristem M. (2003). The evolution, distribution and diversity of endogenous retroviruses. Virus Genes.

[34] Nelson K.E., Peterson J., Gardner M.J., Mungall C., White O., Angiuoli S., Shallom S.J., Selengut J., Rutherford K., Nene V. (2002). Genome sequence of the human malaria parasite Plasmodium falciparum. Nature.

[35] Carlton J.M., Hirt R.P., Silva J.C., Delcher A.L., Schatz M., Zhao Q., Wortman J.R., Bidwell S.L., Alsmark U.C.M., Besteiro S. (2007). Draft Genome Sequence of the Sexually Transmitted Pathogen Trichomonas vaginalis. Science.

[36] Schnable P.S., Page S.E.E.L., Pasternak S., Liang C., Zhang J., Fulton L., Graves T.A., Minx P., Reily A.D., Courtney L. (2012). The B73 Maize Genome: Complexity, Diversity, and Dynamics. Science.

[37] The Arabidopsis Genome Initiative (2000). Analysis of the genome sequence of the flowering plant Arabidopsis thaliana. Nature.

[38] Chalopin D., Naville M., Plard F., Galiana D., Volff J.N. (2015). Comparative analysis of transposable elements highlights mobilome diversity and evolution in vertebrates. Genome Biol. Evol..

[39] Furano A.V., Duvernell D.D., Boissinot S. (2004). L1 (LINE-1) retrotransposon diversity differs dramatically between mammals and fish. Trends Genet..

[40] Boissinot S., Sookdeo A. (2016). The Evolution of Line-1 in Vertebrates. Genome Biol. Evol..

[41] Qian Y., Mancini-DiNardo D., Judkins T., Cox H.C., Brown K., Elias M., Singh N., Daniels C., Holladay J., Coffee B. (2017). Identification of pathogenic retrotransposon insertions in cancer predisposition genes. Cancer Genet..

[42] Green P.M., Bagnall R.D., Waseem N.H., Giannelli F. (2008). Haemophilia A mutations in the UK: Results of screening one-third of the population. Br. J. Haematol..

[43] Hancks D.C., Kazazian H.H. (2016). Roles for retrotransposon insertions in human disease. Mob. DNA.

[44] Kofler R., Betancourt A.J., Schlötterer C. (2012). Sequencing of pooled DNA samples (Pool-Seq) uncovers complex dynamics of transposable element insertions in Drosophila melanogaster. PLoS Genet..

[45] Petrov D.A., Fiston-Lavier A.-S., Lipatov M., Lenkov K., Gonzalez J. (2011). Population Genomics of Transposable Elements in Drosophila melanogaster. Mol. Biol. Evol..

[46] Stritt C., Gordon S.P., Wicker T., Vogel J.P., Roulin A.C. (2018). Recent activity in expanding populations and purifying selection have shaped transposable element landscapes across natural accessions of the mediterranean grass Brachypodium distachyon. Genome Biol. Evol..

[47] Hazzouri K.M., Mohajer A., Dejak S.I., Otto S.P., Wright S.I. (2008). Contrasting patterns of transposable-element insertion polymorphism and nucleotide diversity in autotetraploid and allotetraploid Arabidopsis species. Genetics.

[48] González J., Macpherson J.M., Messer P.W., Petrov D.A. (2009). Inferring the strength of selection in Drosophila under complex demographic models. Mol. Biol. Evol..

[49] Boissinot S., Davis J., Entezam A., Petrov D., Furano A.V. (2006). Fitness cost of LINE-1 (L1) activity in humans. Proc. Natl. Acad. Sci. USA.

[50] Xue A.T., Ruggiero R.P., Hickerson M.J., Boissinot S. (2018). Differential effect of selection against LINE retrotransposons among vertebrates inferred from whole-genome data and demographic modeling. Genome Biol. Evol..

[51] Ruggiero R.P., Bourgeois Y., Boissinot S. (2017). LINE Insertion Polymorphisms Are Abundant but at Low Frequencies across Populations of Anolis carolinensis. Front. Genet..

[52] Quadrana L., Silveira A.B., Mayhew G.F., LeBlanc C., Martienssen R.A., Jeddeloh J.A., Colot V. (2016). The Arabidopsis thaliana mobilome and its impact at the species level. Elife.

[53] Olivares M., Alonso C., López M.C. (1997). The open reading frame 1 of the L1Tc retrotransposon of Trypanosoma cruzi codes for a protein with apurinic-apyrimidinic nuclease activity. J. Biol. Chem..

[54] Conte C., Dastugue B., Vaury C. (2002). Promoter competition as a mechanism of transcriptional interference mediated by retrotransposons. EMBO J..

[55] Langley C.H., Montgomery E.A., Hudson R., Kaplan N., Charlesworth B. (1988). On the role of unequal exchange in the containment of transposable element copy number. Genet. Res..

[56] Dolgin E.S., Charlesworth B. (2008). The effects of recombination rate on the distribution and abundance of transposable elements. Genetics.

[57] Petrov D.A., Aminetzach Y.T., Davis J.C., Bensasson D., Hirsh A.E. (2003). Size matters: Non-LTR retrotransposable elements and ectopic recombination in Drosophila. Mol. Biol. Evol..

[58] Cordaux R., Lee J., Dinoso L., Batzer M.A. (2006). Recently integrated Alu retrotransposons are essentially neutral residents of the human genome. Gene.

[59] Song M., Boissinot S. (2007). Selection against LINE-1 retrotransposons results principally from their ability to mediate ectopic recombination. Gene.

[60] Nam K., Ellegren H. (2012). Recombination drives vertebrate genome contraction. PLoS Genet..

[61] Charlesworth B. (2003). The organization and evolution of the human Y chromosome. Genome Biol..

[62] Boissinot S., Entezam A., Furano A. (2001). V Selection Against Deleterious LINE-1-Containing Loci in the Human Lineage. Mol. Biol..

[63] Montgomery E., Charlesworth B., Langley C. (1987). A test for the role of natural selection in the stabilization of transposable element copy number in a population of Drosophila melanogaster. Genet Res..

[64] Montgomery E.A., Huang S.M., Langley C.H., Judd B.H. (1991). Chromosome rearrangement by ectopic recombination in Drosophila melanogaster: Genome structure and evolution. Genetics.

[65] Le Rouzic A., Boutin T.S., Capy P. (2007). Long-term evolution of transposable elements. Proc. Natl. Acad. Sci. USA.

[66] Charlesworth B., Sniegowski P., Stephan W. (1994). The evolutionary dynamics of repetitive DNA in eukaryotes. Nature.

[67] Ross-Ibarra J., Wright S.I., Foxe J.P., Kawabe A., DeRose-Wilson L., Gos G., Charlesworth D., Gaut B.S. (2008). Patterns of polymorphism and demographic history in natural populations of Arabidopsis lyrata. PLoS ONE.

[68] García Guerreiro M.P., Chávez-Sandoval B.E., Balanyà J., Serra L., Fontdevila A. (2008). Distribution of the transposable elements bilbo and gypsy in original and colonizing populations of Drosophila subobscura. BMC Evol. Biol..

[69] Blass E., Bell M., Boissinot S. (2012). Accumulation and rapid decay of non-LTR retrotransposons in the genome of the three-spine stickleback. Genome Biol. Evol..

[70] Tollis M., Boissinot S. (2013). Lizards and LINEs: Selection and demography affect the fate of L1 retrotransposons in the genome of the green anole (Anolis carolinensis). Genome Biol. Evol..

[71] Lynch M., Conery J.S. (2003). The Origins of Genome Complexity. Science.

[72] Vieira C., Lepetit D., Dumont S., Biémont C. (1999). Wake up of transposable elements following Drosophila simulans worldwide colonization. Mol. Biol. Evol..

[73] Piegu B., Guyot R., Picault N., Roulin A., Saniyal A., Kim H., Collura K., Brar D.S., Jackson S., Wing R.A. (2006). Doubling genome size without polyploidization: Dynamics of retrotransposition-driven genomic expansions in Oryza australiensis, a wild relative of rice. Genome Res..

[74] Manthey J.D., Moyle R.G., Boissinot S. (2018). Multiple and independent phases of transposable element amplification in the genomes of piciformes (woodpeckers and allies). Genome Biol. Evol..

[75] De Boer J.G., Yazawa R., Davidson W.S., Koop B.F. (2007). Bursts and horizontal evolution of DNA transposons in the speciation of pseudotetraploid salmonids. BMC Genomics.

[76] Hellen E.H.B., Brookfield J.F.Y. (2013). The diversity of class II transposable elements in mammalian genomes has arisen from ancestral phylogenetic splits during ancient waves of proliferation through the genome. Mol. Biol. Evol..

[77] Hellen E.H.B., Brookfield J.F.Y. (2013). Transposable element invasions. Mob. Genet. Elements.

[78] Bergman C.M., Bensasson D. (2007). Recent LTR retrotransposon insertion contrasts with waves of non-LTR insertion since speciation in Drosophila melanogaster. Proc. Natl. Acad. Sci. USA.

[79] Haller B.C., Messer P.W. (2019). SLiM 3: Forward Genetic Simulations Beyond the Wright-Fisher Model. Mol. Biol. Evol..

[80] Kent T.V., Uzunović J., Wright S.I. (2017). Coevolution between transposable elements and recombination. Philos. Trans. R. Soc. B Biol. Sci..

[81] Choi K., Zhao X., Kelly K.A., Venn O., Higgins J.D., Yelina N.E., Hardcastle T.J., Ziolkowski P.A., Copenhaver G.P., Franklin F.C.H. (2013). Arabidopsis meiotic crossover hot spots overlap with H2A.Z nucleosomes at gene promoters. Nat. Genet..

[82] Myers S., Bottolo L., Freeman C., McVean G., Donnelly P. (2005). A Fine-Scale Map of Recombination Rates and Hotspots Across the Human Genome. Science.

[83] Hill W.G., Robertson A. (1966). Local effects of limited recombination. Genet. Res..

[84] Felsenstein J. (1974). The evolution advantage of recombination. Genetics.

[85] Kawakami T., Mugal C.F., Suh A., Nater A., Burri R., Smeds L., Ellegren H. (2017). Whole-genome patterns of linkage disequilibrium across flycatcher populations clarify the causes and consequences of fine-scale recombination rate variation in birds. Mol. Ecol..

[86] Jensen-Seaman M.I., Furey T.S., Payseur B.A., Lu Y., Roskin K.M., Chen C.F., Thomas M.A., Haussler D., Jacob H.J. (2004). Comparative recombination rates in the rat, mouse, and human genomes. Genome Res..

[87] Bartolomé C., Maside X., Charlesworth B. (2002). On the abundance and distribution of transposable elements in the genome of Drosophila melanogaster. Mol. Biol. Evol..

[88] Rizzon C., Marais G., Gouy M., Biémont C. (2002). Recombination rate and the distribution of transposable elements in the Drosophila melanogaster genome. Genome Res..

[89] Myers S., Freeman C., Auton A., Donnelly P., McVean G. (2008). A common sequence motif associated with recombination hot spots and genome instability in humans. Nat. Genet..

[90] Campos-Sánchez R., Cremona M.A., Pini A., Chiaromonte F., Makova K.D. (2016). Integration and Fixation Preferences of Human and Mouse Endogenous Retroviruses Uncovered with Functional Data Analysis. PLoS Comput. Biol..

[91] Duret L., Marais G., Biemont C. (2000). Transposons but not retrotransposons are located preferentially in regions of high recombination rate in Caenorhabditis elegans. Genetics.

[92] Csilléry K., Blum M.G.B., Gaggiotti O.E., François O. (2010). Approximate Bayesian Computation (ABC) in practice. Trends Ecol. Evol..

[93] Ågren J.A., Wright S.I. (2011). Co-evolution between transposable elements and their hosts: A major factor in genome size evolution?. Chromosom. Res..

[94] Goodier J.L. (2016). Restricting retrotransposons: A review. Mob. DNA.

[95] Arias J.F., Koyama T., Kinomoto M., Tokunaga K. (2012). Retroelements versus APOBEC3 family members: No great escape from the magnificent seven. Front. Microbiol..

[96] Koito A., Ikeda T. (2013). Intrinsic immunity against retrotransposons by APOBEC cytidine deaminases. Front. Microbiol..

[97] Lindič N., Budič M., Petan T., Knisbacher B.A., Levanon E.Y., Lovšin N. (2013). Differential inhibition of LINE1 and LINE2 retrotransposition by vertebrate AID/APOBEC proteins. Retrovirology.

[98] Yoder J.A., Walsh C.P., Bestor T.H. (1997). Cytosine methylation and the ecology of intragenomic parasites. Trends Genet..

[99] Huda A., Mariño-Ramírez L., Jordan I.K. (2010). Epigenetic histone modifications of human transposable elements: Genome defense versus exaptation. Mob. DNA.

[100] Cheng C., Tarutani Y., Miyao A., Ito T., Yamazaki M., Sakai H., Fukai E., Hirochika H. (2015). Loss of function mutations in the rice chromomethylase OsCMT3a cause a burst of transposition. Plant J..

[101] Van Rij R.P., Berezikov E. (2009). Small RNAs and the control of transposons and viruses in Drosophila. Trends Microbiol..

[102] Prud’homme N., Gans M., Masson M., Terzian C., Bucheton A. (1995). Flamenco, a gene controlling the gypsy retrovirus of Drosophila melanogaster. Genetics.

[103] Goriaux C., Desset S., Renaud Y., Vaury C., Brasset E. (2014). Transcriptional properties and splicing of the flamenco piRNA cluster. EMBO Rep..

[104] Kofler R. (2019). Dynamics of transposable element invasions with piRNA clusters. Mol. Biol. Evol..

[105] Roessler K., Bousios A., Meca E., Gaut B.S. (2018). Modeling Interactions between Transposable Elements and the Plant Epigenetic Response: A Surprising Reliance on Element Retention. Genome Biol. Evol..

[106] Palmer W.H., Hadfield J.D., Obbard D.J. (2018). RNA-Interference Pathways Display High Rates of Adaptive Protein Evolution in Multiple Invertebrates. Genetics.

[107] Simkin A., Wong A., Poh Y.P., Theurkauf W.E., Jensen J.D. (2013). Recurrent and recent selective sweeps in the piRNA pathway. Evolution.

[108] Jacobs F.M.J., Greenberg D., Nguyen N., Haeussler M., Ewing A.D., Katzman S., Paten B., Salama S.R., Haussler D. (2014). An evolutionary arms race between KRAB zinc-finger genes ZNF91/93 and SVA/L1 retrotransposons. Nature.

[109] Haasl R.J., Payseur B.A. (2016). Fifteen years of genomewide scans for selection: Trends, lessons and unaddressed genetic sources of complication. Mol. Ecol..

[110] Miller W.J., McDonald J.F., Nouaud D., Anxolabehere D. (1999). Molecular domestication—More than a sporadic episode in evolution. Genetica.

[111] Jung D., Alt F.W. (2004). Unraveling V(D)J Recombination: Insights into Gene Regulation. Cell.

[112] Oettinger M.A., Schatz D.G., Gorka C., Baltimore D., Oetringer M.A. (1990). RAG-1 and RAG-2, Adjacent Genes That Synergistically Activate V(D)J Recombination. Science.

[113] Kapitonov V.V., Koonin E.V. (2015). Evolution of the RAG1-RAG2 locus: Both proteins came from the same transposon. Biol. Direct.

[114] Pardue M.-L., DeBaryshe P.G. (2011). Retrotransposons that maintain chromosome ends. Proc. Natl. Acad. Sci. USA.

[115] Lee Y.C.G., Leek C., Levine M.T. (2017). Recurrent Innovation at Genes Required for Telomere Integrity in Drosophila. Mol. Biol. Evol..

[116] Zaratiegui M., Vaughn M.W., Irvine D.V., Goto D., Watt S., Bähler J., Arcangioli B., Martienssen R.A. (2011). CENP-B preserves genome integrity at replication forks paused by retrotransposon LTR. Nature.

[117] Gao D., Jiang N., Wing R.A., Jiang J., Jackson S.A. (2015). Transposons play an important role in the evolution and diversification of centromeres among closely related species. Front. Plant Sci..

[118] Capy P., Gasperi G., Biémont C., Bazin C. (2000). Stress and transposable elements: Co-evolution or useful parasites?. Heredity.

[119] Rey O., Danchin E., Mirouze M., Loot C., Blanchet S. (2016). Adaptation to Global Change: A Transposable Element-Epigenetics Perspective. Trends Ecol. Evol..

[120] Kalendar R., Tanskanen J., Immonen S., Nevo E., Schulman A.H. (2000). Genome evolution of wild barley (Hordeum spontaneum) by BARE-1 retrotransposon dynamics in response to sharp microclimatic divergence. Proc. Natl. Acad. Sci. USA.

[121] Feiner N. (2016). Accumulation of transposable elements in *HOX* gene clusters during adaptive radiation of Anolis lizards. Proc. Biol. Sci..

[122] Yang L., Bennetzen J.L. (2009). Distribution, diversity, evolution, and survival of Helitrons in the maize genome. Proc. Natl. Acad. Sci. USA.

[123] Schrader L., Kim J.W., Ence D., Zimin A., Klein A., Wyschetzki K., Weichselgartner T., Kemena C., Stökl J., Schultner E. (2014). Transposable element islands facilitate adaptation to novel environments in an invasive species. Nat. Commun..

[124] Hof A.E.V., Campagne P., Rigden D.J., Yung C.J., Lingley J., Quail M.A., Hall N., Darby A.C., Saccheri I.J. (2016). The industrial melanism mutation in British peppered moths is a transposable element. Nature.

[125] González J., Petrov D.A. (2009). The adaptive role of transposable elements in the Drosophila genome. Gene.

[126] González J., Karasov T.L., Messer P.W., Petrov D.A. (2010). Genome-wide patterns of adaptation to temperate environments associated with transposable elements in Drosophila. PLoS Genet..

[127] Ullastres A., Petit N., González J. (2015). Exploring the phenotypic space and the evolutionary history of a natural mutation in drosophila melanogaster. Mol. Biol. Evol..

[128] Guio L., Barrõn M.G., González J. (2014). The transposable element Bari-Jheh mediates oxidative stress response in Drosophila. Mol. Ecol..

[129] Rech G.E., Bogaerts-Marquez M., Barron M.G., Merenciano M., Villanueva-Canas J.L., Horvath V., Fiston-Lavier A.-S., Luyten I., Venkataram S., Quesneville H. (2018). Stress response, behavior, and development are shaped by transposable element-induced mutations in Drosophila. PLoS Genet..

[130] González J., Macpherson J.M., Petrov D.A. (2009). A recent adaptive transposable element insertion near highly conserved developmental loci in Drosophila melanogaster. Mol. Biol. Evol..

[131] Rishishwar L., Wang L., Wang J., Yi S.V., Lachance J., Jordan I.K. (2018). Evidence for positive selection on recent human transposable element insertions. Gene.

[132] Feschotte C. (2008). Transposable elements and the evolution of regulatory networks. Nat. Rev. Genet..

[133] Lotterhos K.E., Whitlock M.C. (2014). Evaluation of demographic history and neutral parameterization on the performance of FST outlier tests. Mol. Ecol..

[134] Sabeti P.C., Schaffner S.F., Fry B., Lohmueller J., Varilly P., Shamovsky O., Palma A., Mikkelsen T.S., Altshuler D., Lander E.S. (2006). Positive natural selection in the human lineage. Science.

[135] Garud N.R., Messer P.W., Buzbas E.O., Petrov D.A. (2015). Recent Selective Sweeps in North American Drosophila melanogaster Show Signatures of Soft Sweeps. PLoS Genet..

[136] Ferrer-Admetlla A., Liang M., Korneliussen T., Nielsen R. (2014). On detecting incomplete soft or hard selective sweeps using haplotype structure. Mol. Biol. Evol..

[137] McCarroll S.A., Sabeti P.C., Frazer K.A., Varilly P., Fry B., Ballinger D.G., Lohmueller J., Cox D.R., Hostetter E., Hinds D.A. (2007). Genome-wide detection and characterization of positive selection in human populations. Nature.

[138] Gautier M. (2015). Genome-Wide Scan for Adaptive Divergence and Association with Population-Specific Covariates. Genetics.

[139] Rasmussen M.D., Hubisz M.J., Gronau I., Siepel A. (2014). Genome-Wide Inference of Ancestral Recombination Graphs. PLoS Genet..

[140] Schrider D.R., Kern A.D. (2018). Supervised Machine Learning for Population Genetics: A New Paradigm. Trends Genet..

[141] Schrider D.R., Kern A.D. (2017). Machine Learning for Population Genetics: A New Paradigm. bioRxiv.

[142] Schrider D.R., Mendes F.K., Hahn M.W., Kern A.D. (2015). Soft shoulders ahead: Spurious signatures of soft and partial selective sweeps result from linked hard sweeps. Genetics.

[143] Messer P.W., Petrov D.A. (2013). Population genomics of rapid adaptation by soft selective sweeps. Trends Ecol. Evol..

[144] Kern A.D., Schrider D.R. (2018). diploS/HIC: An Updated Approach to Classifying Selective Sweeps. G3.

[145] Lee K.M., Coop G. (2018). Distinguishing Among Modes of Convergent Adaptation Using Population Genomic Data. Genetics.

[146] Sellis D., Callahan B.J., Petrov D.A., Messer P.W. (2011). Heterozygote advantage as a natural consequence of adaptation in diploids. Proc. Natl. Acad. Sci. USA.

[147] Siewert K.M., Voight B.F. (2017). Detecting Long-Term Balancing Selection Using Allele Frequency Correlation. Mol. Biol. Evol..

[148] DeGiorgio M., Lohmueller K.E., Nielsen R. (2014). A model-based approach for identifying signatures of ancient balancing selection in genetic data. PLoS Genet..

[149] Van Oosterhout C. (2009). Transposons in the MHC: The Yin and Yang of the vertebrate immune system. Heredity.

[150] Chen B., Zhang B., Xu L., Li Q., Jiang F., Yang P., Xu Y., Kang L. (2017). Transposable Element-Mediated Balancing Selection at Hsp90 Underlies Embryo Developmental Variation. Mol. Biol. Evol..

[151] van Oosterhout C. (2009). A new theory of MHC evolution: Beyond selection on the immune genes. Proc. Biol. Sci..

[152] Nicod J., Davies R.W., Cai N., Hassett C., Goodstadt L., Cosgrove C., Yee B.K., Lionikaite V., McIntyre R.E., Remme C.A. (2016). Genome-wide association of multiple complex traits in outbred mice by ultra-low-coverage sequencing. Nat. Genet..

[153] Gardner E.J., Lam V.K., Harris D.N., Chuang N.T., Scott E.C., Pittard W.S., Mills R.E., Devine S.E., 1000 Genomes Project Consortium (2017). The Mobile Element Locator Tool (MELT): Population-scale mobile element discovery and biology. Genome Res..

[154] Wen Y.J., Zhang H., Ni Y.L., Huang B., Zhang J., Feng J.Y., Wang S.B., Dunwell J.M., Zhang Y.M., Wu R. (2018). Methodological implementation of mixed linear models in multi-locus genome-wide association studies. Brief. Bioinform..

[155] Rishishwar L., Mariño-Ramírez L., Jordan I.K. (2017). Benchmarking computational tools for polymorphic transposable element detection. Brief. Bioinform..

[156] Kofler R., Gómez-Sánchez D., Schlötterer C. (2016). PoPoolationTE2: Comparative Population Genomics of Transposable Elements Using Pool-Seq. Mol. Biol. Evol..

[157] Fiston-Lavier A.S., Barrón M.G., Petrov D.A., González J. (2015). T-lex2: Genotyping, frequency estimation and re-annotation of transposable elements using single or pooled next-generation sequencing data. Nucleic Acids Res..

[158] Santander C.G., Gambron P., Marchi E., Karamitros T., Katzourakis A., Magiorkinis G. (2017). STEAK: A specific tool for transposable elements and retrovirus detection in high-throughput sequencing data. Virus Evol..

[159] Rahman R., Chirn G.W., Kanodia A., Sytnikova Y.A., Brembs B., Bergman C.M., Lau N.C. (2015). Unique transposon landscapes are pervasive across Drosophila melanogaster genomes. Nucleic Acids Res..

[160] Disdero E., Filée J. (2017). LoRTE: Detecting transposon-induced genomic variants using low coverage PacBio long read sequences. Mob. DNA.

[161] Jiang C., Chen C., Huang Z., Liu R., Verdier J. (2015). ITIS, a bioinformatics tool for accurate identification of transposon insertion sites using next-generation sequencing data. BMC Bioinformatics.

[162] Zhuang J., Wang J., Theurkauf W., Weng Z. (2014). TEMP: A computational method for analyzing transposable element polymorphism in populations. Nucleic Acids Res..

[163] Thung D.T., de Ligt J., Vissers L.E.M., Steehouwer M., Kroon M., de Vries P., Slagboom E.P., Ye K., Veltman J.A., Hehir-Kwa J.Y. (2014). Mobster: Accurate detection of mobile element insertions in next generation sequencing data. Genome Biol..

[164] Wu J., Lee W.P., Ward A., Walker J.A., Konkel M.K., Batzer M.A., Marth G.T. (2014). Tangram: A comprehensive toolbox for mobile element insertion detection. BMC Genomics.

[165] Keane T.M., Wong K., Adams D.J. (2013). RetroSeq: Transposable element discovery from next-generation sequencing data. Bioinformatics.

[166] Chen J., Wrightsman T.R., Wessler S.R., Stajich J.E. (2017). RelocaTE2: A high resolution transposable element insertion site mapping tool for population resequencing. PeerJ.

[167] Nelson M.G., Linheiro R.S., Bergman C.M. (2017). McClintock: An Integrated Pipeline for Detecting Transposable Element Insertions in Whole-Genome Shotgun Sequencing Data. G3.

[168] Seehausen O., Butlin R.K., Keller I., Wagner C.E., Boughman J.W., Hohenlohe P.A., Peichel C.L., Saetre G.-P., Bank C., Brannstrom A. (2014). Genomics and the origin of species. Nat. Rev. Genet..

[169] Butlin R.K., Smadja C.M. (2017). Coupling, Reinforcement, and Speciation. Am. Nat..

[170] Jangam D., Feschotte C., Betrán E. (2017). Transposable Element Domestication As an Adaptation to Evolutionary Conflicts. Trends Genet..

[171] Lindholm A.K., Dyer K.A., Firman R.C., Fishman L., Forstmeier W., Holman L., Johannesson H., Knief U., Kokko H., Larracuente A.M. (2016). The Ecology and Evolutionary Dynamics of Meiotic Drive. Trends Ecol. Evol..

[172] Gardner A., Úbeda F. (2017). The meaning of intragenomic conflict. Nat. Ecol. Evol..

[173] Crespi B., Nosil P. (2013). Conflictual speciation: Species formation via genomic conflict. Trends Ecol. Evol..

[174] Serrato-Capuchina A., Matute D.R. (2018). The role of transposable elements in speciation. Genes.

[175] Daniels S.B., Peterson K.R., Strausbaugh L.D., Kidwell M.G., Chovnik A. (1990). Evidence for horizontal transmission of the P transposable element between Drosophila species. Genetics.

[176] Kidwell M.G. (1979). Hybrid dysgenesis in Drosophila melanogaster: The relationship between the P–M and I–R interaction systems. Genet. Res..

[177] Kimura K., Kidwell M.G. (1994). Differences in P element population dynamics between the sibling species Drosophila melanogaster and Drosophila simulans. Genet. Res..

[178] Yoshitake Y., Inomata N., Sano M., Kato Y., Itoh M. (2018). The P element invaded rapidly and caused hybrid dysgenesis in natural populations of Drosophila simulans in Japan. Ecol. Evol..

[179] Hill T., Schlötterer C., Betancourt A.J. (2016). Hybrid Dysgenesis in Drosophila simulans Associated with a Rapid Invasion of the P-Element. PLoS Genet..

[180] Kofler R., Hill T., Nolte V., Betancourt A.J., Schlötterer C. (2015). The recent invasion of natural *Drosophila simulans* populations by the P-element. Proc. Natl. Acad. Sci. USA.

[181] O’Neill M.J., O’Neill R.J. (2018). Sex chromosome repeats tip the balance towards speciation. Mol. Ecol..

[182] Brown J.D., O’Neill R.J. (2010). Chromosomes, Conflict, and Epigenetics: Chromosomal Speciation Revisited. Annu. Rev. Genom. Hum. Genet..

[183] Ellison C., Bachtrog D. (2013). Dosage Compensation via Transposable Element Mediated Rewiring of a Regulatory Network. Science.

[184] Conrad T., Akhtar A. (2012). Dosage compensation in Drosophila melanogaster: Epigenetic fine-tuning of chromosome-wide transcription. Nat. Rev. Genet..

[185] Gay L., Crochet P.-A., Bell D.A., Lenormand T. (2008). Comparing clines on molecular and phenotypic traits in hybrid zones: a window on tension zone models. Evolution.

[186] Lesecque Y., Glémin S., Lartillot N., Mouchiroud D., Duret L. (2014). The Red Queen Model of Recombination Hotspots Evolution in the Light of Archaic and Modern Human Genomes. PLoS Genet..

[187] Bierne N., Welch J., Loire E., Bonhomme F., David P. (2011). The coupling hypothesis: Why genome scans may fail to map local adaptation genes. Mol. Ecol..

[188] Andrew R.L., Bernatchez L., Bonin A., Buerkle C.A., Carstens B.C., Emerson B.C., Garant D., Giraud T., Kane N.C., Rogers S.M. (2013). A road map for molecular ecology. Mol. Ecol..

[189] Li J., Li H., Jakobsson M., Li S., SjÖdin P., Lascoux M. (2012). Joint analysis of demography and selection in population genetics: Where do we stand and where could we go?. Mol. Ecol..

[190] Orozco-terWengel P. (2016). The devil is in the details: The effect of population structure on demographic inference. Heredity.

[191] Sattath S., Elyashiv E., Kolodny O., Rinott Y., Sella G. (2011). Pervasive adaptive protein evolution apparent in diversity patterns around amino acid substitutions in drosophila simulans. PLoS Genet..

[192] Suh A., Smeds L., Ellegren H. (2018). Abundant recent activity of retrovirus-like retrotransposons within and among flycatcher species implies a rich source of structural variation in songbird genomes. Mol. Ecol..

